# Finding all maximal perfect haplotype blocks in linear time

**DOI:** 10.1186/s13015-020-0163-6

**Published:** 2020-02-10

**Authors:** Jarno Alanko, Hideo Bannai, Bastien Cazaux, Pierre Peterlongo, Jens Stoye

**Affiliations:** 1 Department of Computer Science, University of Helsinki, Helsinki, Finland; 2 Department of Informatics, Kyushu University, Fukuoka, Japan; 3 Inria, CNRS, Irisa, Univ. Rennes, Rennes, France; 4 Faculty of Technology and Center for Biotechnology (CeBiTec), Bielefeld University, Bielefeld, Germany

**Keywords:** Population genomics, Selection coefficient, Haplotype block, Positional Burrows–Wheeler Transform

## Abstract

Recent large-scale community sequencing efforts allow at an unprecedented level of detail the identification of genomic regions that show signatures of natural selection. Traditional methods for identifying such regions from individuals’ haplotype data, however, require excessive computing times and therefore are not applicable to current datasets. In 2019, Cunha et al. (Advances in bioinformatics and computational biology: 11th Brazilian symposium on bioinformatics, BSB 2018, Niterói, Brazil, October 30 - November 1, 2018, Proceedings, 2018. 10.1007/978-3-030-01722-4_3) suggested the *maximal perfect haplotype block* as a very simple combinatorial pattern, forming the basis of a new method to perform rapid genome-wide selection scans. The algorithm they presented for identifying these blocks, however, had a worst-case running time quadratic in the genome length. It was posed as an open problem whether an optimal, linear-time algorithm exists. In this paper we give two algorithms that achieve this time bound, one conceptually very simple one using suffix trees and a second one using the positional Burrows–Wheeler Transform, that is very efficient also in practice.

## Introduction and background

As a result of the technological advances that went hand in hand with the genomics efforts of the last decades, today it is possible to experimentally obtain and study the genomes of large numbers of individuals, or even multiple samples from an individual. For instance, the *National Human Genome Research Institute* and the *European Bioinformatics Institute* have collected more than 3500 genome-wide association study publications in their *GWAS Catalog* [1].

Probably the most prominent example of large-scale sequencing projects is the *1000 Genomes Project* (now *International Genome Sample Resource*, IGSR), initiated with the goal of sequencing the genomes of more than one thousand human individuals to identify 95% of all genomic variants in the population with allele frequency of at least 1% (down toward 0.1% in coding regions). The final publications from phase 3 of the project report about genetic variations from more than 2500 genomes [2, 3].

Recently, several countries announced large-scale national research programs to capture the diversity of their populations, while some of these efforts started already more than 20 years ago. Since 1996 Iceland’s deCODE company is mining Icelanders’ genetic and medical data for disease genes. In 2015, deCODE published insights gained from sequencing the whole genomes of 2636 Icelanders [4]. *Genome of the Netherlands* (GoNL) is a whole genome sequencing project aiming to characterize DNA sequence variation in the Dutch population using a representative sample consisting of 250 trio families from all provinces in the Netherlands. In 2016, GoNL analysed whole genome sequencing data of 769 individuals and published a haplotype-resolved map of 1.9 million genome variants [5]. Similar projects have been established in larger scale in the UK: Following the *UK10K* project for identifying rare genetic variants in health and disease (2010–2013), *Genomics England* was set up in late 2012 to deliver the 100,000 Genomes Project [6]. This flagship project has by now sequenced 100,000 whole genomes from patients and their families, focusing on rare diseases, some common types of cancer, and infectious diseases. The scale of these projects is culminating in the US federal *Precision Medicine Initiative*, where the NIH is funding the *All of Us* research program^1^ to analyze genetic information from more than 1 million American volunteers. Even more extreme suggestions go as far as to propose “to sequence the DNA of all life on Earth”^2^.

The main motivation for the collection of these large and comprehensive data sets is the hope for a better understanding of genomic variation and how variants relate to health and disease, but basic research in evolution, population genetics, functional genomics and studies on demographic history can also profit enormously.

One important approach connecting evolution and functional genomics is the search for genomic regions under natural selection based on population data. The *selection coefficient* [7] is an established parameter quantifying the relative fitness of two genetic variants. Unfortunately, haplotype-based methods for estimating selection coefficients have not been designed with the massive genome data sets available today in mind, and may therefore take prohibitively long when applied to large-scale population data. In view of the large population sequencing efforts described above, methods are needed that—at similar sensitivity—scale to much higher dimensions.

Only recently a method for the fast computation of a genome-wide selection scan has been proposed that can be computed quickly even for large datasets [8]. The method is based on a very simple combinatorial string pattern, *maximal perfect haplotype blocks*. Although considerably faster than previous methods, the running time of the algorithm presented in that paper is not optimal, as it takes $O(kn^2)$ time in order to find all maximal perfect haplotype blocks in *k* genomes of length *n* each. This is sufficient to analyse individual human chromosomes on a laptop computer, for datasets of the size of the 1000 Genomes Project (thousands of genomes and millions of variations). However, with the larger datasets currently underway and with higher resolution it will not scale favourably. More efficient methods are therefore necessary and it was phrased as an open question whether there exists a linear-time algorithm to find all maximal perfect haplotype blocks.

In this paper we settle this open problem affirmatively. More specifically, after some basic definitions in “Basic definitions” section we present in “Linear-time method I: based on suffix trees” and “Linear-time method II: based on the positional BWT” sections two new algorithms for finding all maximal perfect haplotype blocks in optimal time. The latter of these two algorithms is then experimentally compared to the one from [8] in “Empirical evaluation” section, proving its superiority in running time by a factor of about 5 and memory usage by up to two orders of magnitude for larger data sets. “Conclusion” section concludes the paper.

This paper is an extended version of the preliminary work presented in [9]. Source code and test data are available from https://gitlab.com/bacazaux/haploblocks.

## Basic definitions

The typical input to genome-wide selection studies is a set of haplotype-resolved genomes, or *haplotypes* for short. Clearly, for a given set of haplotypes only those sites are of interest where there is variation in the genomes. Therefore, formally, we consider as input to our methods a *k* × *nhaplotype matrix* where each of the *k* rows corresponds to one haplotype and each of the *n* columns corresponds to one variable genetic site.

Most methods distinguish only between ancestral and derived allele, reflecting the fact that most sites are biallelic. Therefore the entries in a haplotype matrix are often considered binary where the ancestral allele is encoded by 0 and the derived allele is encoded by 1. However, the computational problem and its solutions considered in this paper do not depend on this restriction and instead are applicable to any type of sequence over a constant-size alphabet $\Sigma$.

The concept of a maximal perfect haplotype block as defined in [8] is the following, where *s*[*i*, *j*] denotes the substring of a string *s* from position *i* to position *j* and $S|_K$ denotes the elements of an ordered set *S* restricted to index set *K*:

### **Definition 1**

Given *k* sequences $S = (s_1,\ldots ,s_k)$ of the same length *n* (representing the rows of a haplotype matrix), a *maximal perfect haplotype block* is a triple (*K*, *i*, *j*) with $K \subseteq \{1,\ldots ,k\}$, $\vert K \vert \ge 2$ and $1 \le i \le j \le n$ such that

1. $s[i,j] = t[i,j]$ for all $s,t \in S|_K$*(equality)*,
2. $i = 1$ or $s[i-1] \ne t[i-1]$ for some $s,t \in S|_K$*(left-maximality)*,
3. $j = n$ or $s[j+1] \ne t[j+1]$ for some $s,t \in S|_K$*(right-maximality)*, and
4. $\not \exists K' \subseteq \{1,\ldots ,k\}$ with $K \subset K'$ such that $s[i,j] = t[i,j]$ for all $s,t \in S|_{K'}$*(row-maximality)*.

**Fig. 1:**
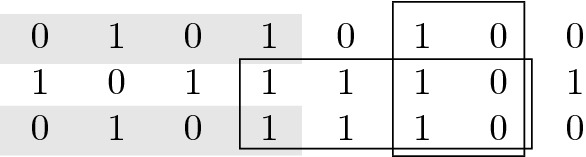
Illustration of Definition 1. A binary $3 \times 8$ haplotype matrix with three maximal perfect haplotype blocks $(\{1,3\},1,4)$, $(\{2,3\},4,7)$ and $(\{1,2,3\},6,7)$ highlighted. (The example contains additional maximal perfect haplotype blocks that are not shown.)

Definition 1 is illustrated in Fig. 1.

In Cunha et al. [8] it was shown that the number of maximal perfect haplotype blocks is *O*(*kn*), while the algorithm presented there takes $O(kn^2)$ time to find all blocks. It is based on the observation that branching vertices in the trie $T_p$ of the suffixes of the input sequences starting at position *p* correspond to right-maximal and row-maximal blocks, while left-maximality can be tested by comparing $T_p$ and $T_{p-1}$. In the next two sections we show how this running time can be improved.

## Linear-time method I: based on suffix trees

In this section, we present our first algorithm to find all maximal perfect haplotype blocks in linear time. This solution is purely theoretical, it would likely require large amounts of memory while being slow in practice. However, it demonstrates the connection to the concept of maximal repeats in strings. We recall from [10, Section 7.12] that a *maximal repeat* is a substring occurring at least twice in a string or a set of strings and such that it cannot be extended to the left or to the right without losing occurrences.

Let $\mathbb {S} = s_1\$_1s_2\$_2\ldots s_k\$_k$, with the $\$_i$ being *k* different characters absent from the original alphabet $\Sigma$. The key point is that any maximal perfect haplotype block in *S* is a maximal repeat in $\mathbb {S}$. The opposite is not true: In a maximal perfect haplotype block, all occurrences of the repeat are located at the same position of each sequence of *S* (equality condition in Definition 1), while this constraint does not exist for maximal repeats in $\mathbb {S}$.

Nevertheless, finding all maximal perfect haplotype blocks in *S* can be performed by computing all maximal repeats in $\mathbb {S}$, while keeping only those whose occurrences are located at the same positions over all $s_i$ in which they occur. This can be done by performing the following procedure^3^:

1. “Decorate” each sequence $s_i \in S$ to create $s_i^+=\alpha _0s_i[1]\alpha _1s_i[2]\alpha _2\ldots s_i[n]\alpha _n$, where the *index characters*$\alpha _0, \alpha _1, \ldots , \alpha _n$ are $n+1$ symbols from an alphabet $\Sigma '$, disjoint from the original alphabet $\Sigma$.
2. Find in $\mathbb {S}^+ = s_1^+\$_1s_2^+\$_2\ldots s_k^+\$_k$ all maximal repeats.
3. Any maximal repeat $r = \alpha _pr_1\alpha _{p+1}r_2\alpha _{p+2}\ldots r_\ell \alpha _{p+\ell }$ in $\mathbb {S}^+$ with $\ell \ge 1$ corresponds to a maximal perfect haplotype block of length $\ell$, starting at position $p+1$ in the input sequences from *S*.

The key idea here is that the index characters impose that each maximal repeat occurrence starts at the same position in all sequences and, as a consequence, ensure that all occurrences occur in distinct sequences from *S*.

Hence any maximal repeat $r = \alpha _pr_1\alpha _{p+1}\ldots r_\ell \alpha _{p+\ell }$ defines a unique maximal perfect haplotype block $(K,p+1,p+\ell )$. The value |*K*| is the number of occurrences of *r*. Also the set *K* can be derived from occurrence positions of *r* in $\mathbb {S}^+$, as any position in *r* corresponds to a unique position in $\mathbb {S}$. We prefer to omit useless technical details here.

The maximal repeat occurrences in $\mathbb {S}^+$ may be found using a suffix tree, constructed in time linear with respect to the size of the input data *O*(*kn*), even for large integer alphabets [12], as we have here. The maximal repeat detection is also linear with the size of the input data [10, Section 7.12.1]. Therefore the overall time complexity is *O*(*kn*).

## Linear-time method II: based on the positional BWT

Here we present our second algorithm to find all maximal perfect haplotype blocks in linear time. It works by scanning the haplotype matrix column by column while maintaining the positional Burrows–Wheeler Transform (pBWT) [13] of the current column. For simplicity of presentation we assume that all rows of the haplotype matrix *S* are distinct. Recall that the pBWT of *S* consists of a pair of arrays for each column of *S*: For each *l*, $1\le l\le n$, we have arrays $a_l$ and $d_l$ of length *k* such that the array $a_l$ is a permutation of the elements in the set $\{1,2,\ldots ,k\}$ with $S\left[ a_l[1]\right] [1,l] \le \cdots \le S\left[ a_l[k]\right] [1,l]$ colexicographically (i.e. right-to-left lexicographically) sorted, and the array $d_l$ indicates the index from which the current and the previous rows coincide. Formally, $d_l[1] = l+1$ and for all *r*, $1 < r \le k$, we have $d_l[r] = 1 + \max \{j \in [1,l] : S\left[ a_l[r]\right] [j] \ne S\left[ a_l[r-1]\right] [j]\}.$ Further let us denote by $a_l^{-1}$ the inverse permutation of $a_l$. For readers familiar with string processing terminology, the arrays $a_l$ and $a_l^{-1}$ are analogous to the suffix array and the inverse suffix array, respectively, while the arrays $d_l$ are analogous to the LCP array.

Conditions 1, 2 and 4 (equality, left-maximality and row-maximality) of Definition 1 can be stated in terms of the arrays $a_l$ and $d_l$ as follows.

### **Definition 2**

A quadruple (*i*, *j*; *x*, *y*) with $1\le i\le j\le n$ and $1\le x<y\le k$ is called an *available block* if the following holds:

- $d_j[r] \le i$ for all $r \in [x+1,y]$ (equality),
- there exists at least one $r \in [x+1,y]$ such that $d_j[r] = i$ (left-maximality), and
- ($x = 1$ or $d_j[x] > i$) and ($y = k$ or $d_j[y+1] > i$) (row-maximality).

The interval [*x*, *y*] of an available block (*i*, *j*; *x*, *y*) is called the *colexicographic range* of the block.

### **Lemma 1**

*Suppose we have a maximal perfect haplotype block* (*K*, *i*, *j*), *then the set*$\{a_j^{-1}[r] \mid r \in K\}$*must be a contiguous range* [*x*, *y*] *of indices such that* (*i*, *j*; *x*, *y*) *is an available block.*

### Proof

This necessary condition follows immediately from Definitions 1 and 2 and the definition of the pBWT (arrays $a_l$ and $d_l$). $\square$

**Fig. 2:**
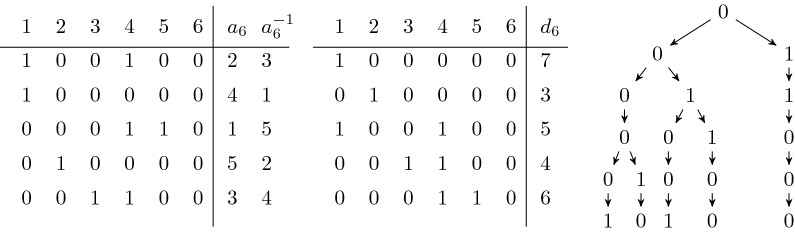
Available blocks. *Left:* an example of a haplotype matrix up to column 6 with the two arrays $a_6$ and $a_6^{-1}$ on the right. *Center:* the colexicographically sorted rows and the array $d_6$ listed on the right. *Right:* the trie of the reverses of the rows of the matrix. For example, the block $(\{1,2,4,5\},5,6)$ is available because $a_6^{-1}(1) = 3$, $a_6^{-1}(2) = 1$, $a_6^{-1}(4) = 2$, $a_6^{-1}(5) = 4$ is the consecutive range $[x,y] = [1,4]$, we have $d_6[r] \le 5$ for all $r \in [1+1,4]$ with $d_6[3] = 5$, and we have $x=1$ and $d_6[4+1] = 6 > 5$. The repeat in the block is 00, and we see it is a branching node in the trie on the right

Let us consider the set $B_l$ of available blocks ending at column *l*. We have that $|B_l| \le k$ because each available block corresponds to a distinct branching node in the trie of the reverses of $\{S[1][1,l], \ldots , S[k][1,l]\}$, and the number of branching nodes in the trie is bounded from above by the number of leaves *k*. The branching nodes of the trie can be enumerated in *O*(*k*) time by using a standard algorithm [14] for enumerating LCP intervals of the LCP array of the trie, $LCP_l[r] = l - d_l[r] + 1$. This gives us the colexicographic ranges [*x*, *y*] of all available blocks in $B_l$. An example is shown in Fig. 2.

The only thing left is to show how to check the right-maximality property of an available block. The following lemma gives the sufficient condition for this:

### **Lemma 2**

*An available block* (*i*, *j*; *x*, *y*) *corresponds to a maximal haplotype block* (*K*, *i*, *j*) *if and only if*$j = n$*or*$|\{S[a[r]][j+1] : r \in [x,y]\}| > 1$.

### Proof

If $j=n$, right-maximality according to Definition 1 holds trivially. If $j<n$, right-maximality requires that there are two rows $s,t \in S|_K$ for which $s[j+1] \not = t[j+1]$. Since all rows *s*, *t* qualifying for this condition are within the colexicographic range [*x*, *y*] of our available block, the statement follows immediately. $\square$

To check the condition of Lemma 2 in constant time for $j \ne n$, we build a bit vector $V_j$ such that $V_j[1] = 1$ and $V_j[r] = 1$ if and only if $S[a_j[r]][j+1] \ne S[a_j[r-1]][j+1]$. Now the block is right-maximal if and only if $V_j[x+1,y]$ contains at least one 1-bit. We can build a vector of prefix sums of $V_j$ to answer this question in constant time.

### Time and space complexity

We assume the *column stream model*, where we can stream the haplotype matrix column by column. We can thus build the arrays $d_l$, $a_l$ and $a_l^{-1}$ on the fly column by column [13], and also easily build the required prefix sums of arrays $V_l$ from these. The time is *O*(*nk*), since each of the *n* columns takes *O*(*k*) time to process. The algorithm needs to keep in memory only the data for two adjacent columns at a time, so in space *O*(*k*) we can report the colexicographic ranges of all maximal blocks ending in each column $l \in [1,n]$. If the colexicographic range of a block at column *l* is [*x*, *y*], then the rows in the original haplotype matrix are $a_l[x], a_l[x+1], \ldots , a_l[y]$. There are *O*(*nk*) blocks and *O*(*k*) rows per block, so the time to report all rows explicitly is $O(nk^2)$. In fact, a sharper bound that can also easily be achieved is $O(nk+z)$ where $z \in O(nk^2)$ is the size of the output. Alternatively, we can store a complete representation of the answer taking *O*(*nk*) space by storing all the $a_l$ arrays and the colexicographic ranges of the maximal perfect blocks for each column, from which we can readily report all rows in any maximal perfect block in constant time per row.

## Empirical evaluation

Since the algorithm of “Linear-time method I: based on suffix trees” section is mostly of theoretical interest, we evaluate only the pBWT-based algorithm presented in “Linear-time method II: based on the positional BWT” section. The source code is available from https://gitlab.com/bacazaux/haploblocks. As a baseline for comparison we use the implementation of the trie-based algorithm by Cunha et al. [8], available from the same gitlab site. The experiments were run on a machine with an Intel Xeon E5-2680 v4 2.4 GHz CPU, which has a 35 MB Intel SmartCache. The machine has 256 gigabytes of memory at a speed of 2400MT/s. The code was compiled with g++ using the -Ofast optimization flag.

Our test data consists of chromosomes 2, 6 and 22 from phase three of the 1000 Genomes Project [2], which provides whole-genome sequences of 2504 individuals from multiple populations worldwide. We preprocessed the data by extracting all biallelic SNPs from the provided VCF files^4^ and converting them to a binary haplotype matrix using our own program vcf2bm, also available from https://gitlab.com/bacazaux/haploblocks.

Our implementation has a user-defined parameter allowing to adjust the minimum size of a reported maximal perfect haplotype block (*K*, *i*, *j*), where *size* is defined as the width ($j-i+1$) times the number of rows (|*K*|) in the block. Table 1 shows the running times and memory usage of our implementation on the different chromosomes and for different settings of the minimum block size parameter. The larger the minimum block size, the faster the algorithm is, because there are less blocks to report. In general, it takes only a few minutes to process a complete human chromosome. Locating all 323,163,970 blocks of minimum size $10^6$ in all 22 human autosomes (non-sex chromosomes) took in total 4 h and 26 min with a memory peak of 12.8 MB (data not shown).

**Table 1:** Running times and memory usage of our pBWT-based implementation

Data set	#lines	#columns	Min block size	Time	Memory (MB)	#blocks
chr. 22	5008	1,055,454		4 min 54 s	12.8	148,613,645
chr. 22	5008	1,055,454	500,000	3 min 50 s	12.8	16,076,453
chr. 22	5008	1,055,454	1,000,000	3 min 40 s	12.8	2,228,762
chr. 22	5008	1,055,454	2,000,000	3 min 43 s	12.8	4779
chr. 6	5008	4,800,101		19 min 42 s	12.8	624,689,548
chr. 6	5008	4,800,101	500,000	17 min 20 s	12.8	89,840,467
chr. 6	5008	4,800,101	1,000,000	16 min 30 s	12.8	11,388,982
chr. 6	5008	4,800,101	2,000,000	16 min 36 s	12.8	5585
chr. 2	5008	6,786,300		31 min 57 s	12.8	946,717,897
chr. 2	5008	6,786,300	500,000	25 min 06 s	12.8	160,094,115
chr. 2	5008	6,786,300	1,000,000	23 min 24 s	12.8	25,533,314
chr. 2	5008	6,786,300	2,000,000	23 min 18 s	12.8	120,243

Note that in our streaming implementation the memory usage is dominated by the number of haplotypes times the buffer size, and therefore is essentially constant in this study

Table 2 shows a comparison of our implementation to the trie-based implementation from [8]. Our implementation is about 5 times faster on all datasets, and the memory consumption is up to 93 times smaller.

**Table 2:** Comparison of the trie-based implementation from [8] and our pBWT-based implementation with minimum block size $10^6$

Data set	trie		pBWT	
	Time	Memory	Time	Memory (MB)
chr. 22	17 min 08 s	927.8 MB	3 min 40 s	12.8
chr. 6	1 h 34 min 34 s	3.23 GB	16 min 30 s	12.8
chr. 2	2 h 07 min 21 s	4.46 GB	23 min 24 s	12.8

**Fig. 3:**
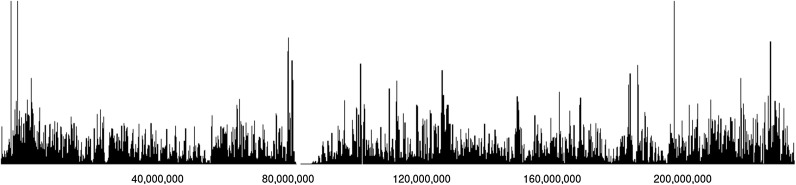
Selection scan for human chromosome 2. Shown is for each position of the chromosome the largest maximum likelihood estimate derived from any maximal perfect haplotype block overlapping that locus. It is easy to spot potential regions of high selection. The centromere, located around 93 Mbp, shows no signal as sequencing coverage is low here and no SNPs could be called

It is now easy to apply the method for estimating a local selection coefficient from the size of maximal perfect haplotype blocks covering a certain genomic region presented in [8]. This method estimates the likelihood of observing a haplotype block for a given selection coefficient *s* and the time *t* since the onset of selection following an approach presented by Chen et al. [15]. Therefore, chromosome-wide selection scans indicating the loci of maximum selection, as shown in Fig. 3 for the complete human chromosome 2 (size parameter $10^6$), can now be generated in less than half an hour.

## Conclusion

In this paper we presented two algorithms that are able to find all maximal perfect haplotype blocks in a haplotype matrix of size $k \times n$ in linear time *O*(*kn*). In particular the second method, based on the positional Burrows–Wheeler Transform, performs also extremely well in practice, as it allows for a streaming implementation with extremely low memory footprint.

While an initial implementation of the method is available from https://gitlab.com/bacazaux/haploblocks, a user-friendly software combining the algorithm presented here with the computation of the selection coefficient suggested in [8] remains to be developed.

## Data Availability

Source code and test data are available from https://gitlab.com/bacazaux/haploblocks.

## Footnotes

1 http://www.allofus.nih.gov.

2 Biologists propose to sequence the DNA of all life on Earth, by Elizabeth Pennisi. Science News, Feb. 24, 2017. https://doi.org/10.1126/science.aal0824.

3 Note that a similar procedure has been described by Lunter [11], where also a connection to the positional Burrows–Wheeler Transform is mentioned.

4 ftp://ftp.1000genomes.ebi.ac.uk/vol1/ftp/release/20130502/.
